# A novel oridonin analogue, CYD0682, suppresses breast cancer growth, angiogenesis, and metastasis by inhibiting the ANGPTL4/MAPK signaling axis

**DOI:** 10.1016/j.gendis.2025.101681

**Published:** 2025-05-08

**Authors:** Xiaobin Mai, Le Wang, Juan Tu, Jialin Li, Jun Li, Yaping Zhan, Pei Tang, Ying Wang, Yan Wang, Lingyun Zheng, Qianqian Zhang, Jiangchao Li, Xiong Li, Lijing Wang, Jia Zhou, Cuiling Qi

**Affiliations:** a School of Basic Medical Sciences, Guangdong Pharmaceutical University, Guangzhou, Guangdong, 510006, China; b Chemical Biology Program, Department of Pharmacology and Toxicology, University of Texas Medical Branch, Galveston, TX 77555, United States

Breast cancer is one of the most common cancers and the leading cause of cancer-related deaths among women due to the diagnostic delay and failure of treatment.^1^ Despite the significant progress made in developing therapeutic strategies for breast cancer, effective treatment of breast cancer, particularly aggressive triple-negative breast cancer, remains lacking. Angiogenesis is a crucial risk factor for breast cancer metastasis and a predictor of poor prognosis. Thus, developing novel agents capable of suppressing tumor angiogenesis offers a promising approach for breast cancer treatment. Oridonin, the major active ingredient of the traditional Chinese medicinal herb, exhibits anti-cancer activity by inhibiting tumor-induced angiogenesis.^2^ Nevertheless, the therapeutic potential of oridonin is limited due to its rapid plasma clearance and limited potency. Various novel oridonin analogues have been designed and chemically synthesized by modifying their A, B, and D rings to achieve an agent with better anti-cancer efficacy and lower toxicity than oridonin.^3^ Chick embryo chorioallantoic membrane (CAM) and yolk sac membrane (YSM) models were commonly utilized to study tumor-induced angiogenesis. To discover the promising anti-angiogenic agents, we screened novel oridonin analogues synthesized in-house using the CAM and YSM models. Ultimately, four molecules were discovered to show the potential anti-angiogenic ability in the CAM (Table S1). Among them, CYD0682 was identified as a promising anti-angiogenic drug candidate in which a double bond was introduced at the 1,2-positions of the A-ring by removal of the hydroxy group on this ring to enhance the drug-like properties such as lipophilicity and cell permeability (Table S1).^4^ Angiopoietin-like protein 4 (ANGPTL4), a member of the ANGPTL family, is closely correlated with tumor growth, metastasis, and angiogenesis. Furthermore, ANGPTL4 down-regulation can promote the invasion and metastasis of colorectal cancer via the extracellular signal-regulated kinase (ERK) signaling pathway.^5^

We first examined the effect of CYD0682 on angiogenesis of CAM and found that CYD0682 (its chemical structure is shown in Fig. 1B) significantly reduced the blood vessel density of CAM in a concentration dependent manner (Fig. 1C, F) and had a stronger ability to inhibit angiogenesis of CAM than oridonin (its chemical structure is shown in Fig. 1A), which is the positive control compound for CYD0682 (Fig. 1C, F). The YSM model was further used to confirm the role of CYD0682 in angiogenesis. Images of the vascular plexus of the YSM within the plastic rings were taken at 0 h and 24 h (Fig. 1D). CYD0682 significantly attenuated the blood vessel density of YSM in a concentration-dependent manner (Fig. 1G). Excitingly, CYD0682 (1.0 μg) significantly decreased the blood vessel density of YSM as compared with oridonin (1.0 μg), demonstrating that CYD0682 showed a stronger ability to inhibit angiogenesis than oridonin (Fig. 1G). To further determine the anti-angiogenic properties of CYD0682, a rat aortic ring assay was performed. Vessel sprouting of rat aortic ring was photographed during the 7 days after treatment with dimethylsulfoxide (DMSO), oridonin, or CYD0682 (Fig. S1A). CYD0682 or oridonin significantly decreased the number of blood vessels sprouting from the aortic rings, and the number of sprouting blood vessels treated with CYD0682 was significantly reduced compared with that in the oridonin-treated aortic rings (Fig. S1B). These results indicate that CYD0682 significantly reduces angiogenesis, and the inhibitory effect on angiogenesis is significantly better than that of oridonin.

**Figure 1:**
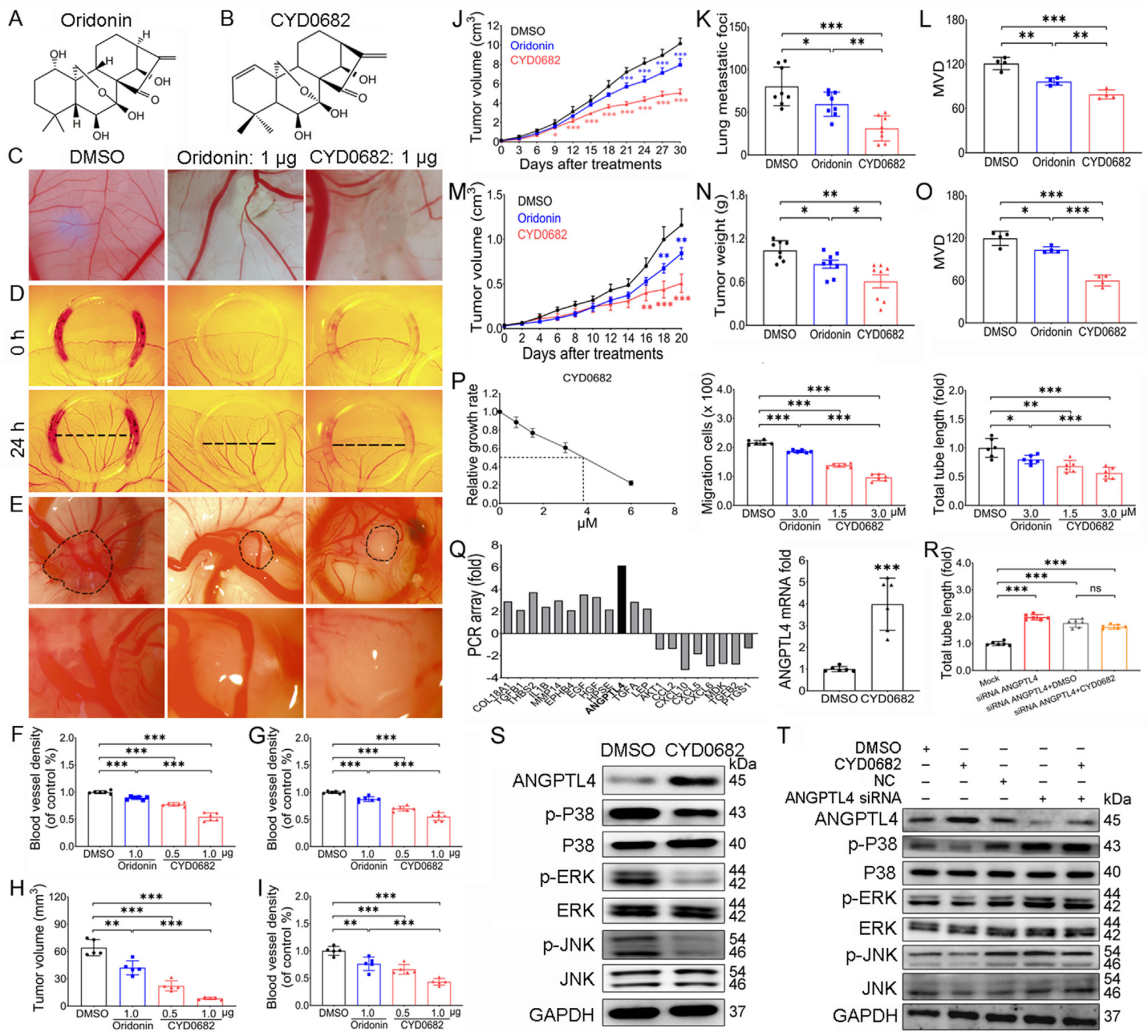
CYD0682, an oridonin analogue, significantly suppresses breast cancer growth and metastasis as well as angiogenesis via regulation of ANGPTL4/MAPK signaling pathway in the vascular endothelial cells. **(A, B)** The chemical structures of oridonin (A) and CYD0682 (B). **(C)** The pictures of the vascular plexus of chicken embryos in each group after 48 h of treatment with oridonin (1.0 μg), CYD0682 (1.0 μg), or DMSO in the CAM model. **(D)** The pictures of the vascular beds of the YSM within plastic rings at 0 h and 24 h after treatment with CYD0682, oridonin, or DMSO. **(E)** Representative images of the blood vessels of breast cancer on CAM treated with DMSO, oridonin, or CYD0682. The panels below indicate the higher magnification of the black dotted frames. The black dotted frames display the tumor. **(F)** The relative blood vessel density of the CAM treated with CYD0682 (0.5 or 1.0 μg), oridonin (1.0 μg), or DMSO. **(G)** The bar chart shows the relative blood vessel density statistics of CYD0682 (0.5 or 1.0 μg), oridonin (1.0 μg), or DMSO for 24 h. **(H)** CYD0682 significantly inhibited breast cancer growth on CAM. **(I)** CYD0682 significantly attenuated breast cancer-induced angiogenesis on CAM. **(J**–**L)** CYD0682 (7.5 mg/kg), oridonin (7.5 mg/kg), or DMSO was injected into nine-week-old MMTV-PyMT mice once every 3 days for 30 days. The long and short diameters of tumors were measured every other day. There were smaller tumors in the CYD0682-treated or oridonin-treated mice as compared with the DMSO-treated mice. Furthermore, CYD0682-treated mice grew smaller tumors than did the oridonin-treated mice (J). There were fewer metastatic foci on the lung surfaces of the CYD0682-treated or oridonin-treated mice as compared with the DMSO-treated mice, and CYD0682-treated mice had fewer metastatic foci than did the oridonin-treated mice (K). Compared with DMSO-treated tumor tissues, there were fewer CD31-positive blood vessels in oridonin- and CYD0682-treated tumor tissues (L). **(M**–**O)** Breast cancer-bearing nude mice were intraperitoneally injected with DMSO, oridonin (7.5 mg/kg), or CYD0682 (7.5 mg/kg) for 20 days. The long and short diameters of tumors were measured every other day. There were smaller tumors in the CYD0682-treated or oridonin-treated mice as compared with the DMSO-treated mice. Importantly, CYD0682-treated mice grew smaller tumors than did the oridonin-treated mice (M). CYD0682-treated or oridonin-treated breast cancer-bearing mice developed lighter tumors than did DMSO-treated mice. Importantly, CYD0682-treated mice grew lighter tumors than did the oridonin-treated mice (N). Oridonin and CYD0682 significantly suppressed tumor-induced angiogenesis. There was lower microvessel density (MVD) in the CYD0682-treated breast cancer tissues as compared with the oridonin-treated tissues (O). **(P)** CYD0682 significantly inhibited human umbilical vein endothelial cell (HUVEC) proliferation in a dose-dependent manner in an MTT assay. The cell migration was significantly suppressed by CYD0682 (1.5 μM), and CYD0682 (3 μM) significantly suppressed HUVEC migration as compared with oridonin (3 μM). The effect of CYD0682 on capillary-like tube formation was calculated using the tube formation assay. The bar chart displayed the tube formation statistics of DMSO, oridonin (3 μM), or CYD0682 (1.5 μM or 3 μM). **(Q)** ANGPTL4 expression was up-regulated in HUVECs treated with CYD0682. **(R)** ANGPTL4 silencing significantly promoted HUVEC tube formation. Furthermore, the ability of ANGPTL4-silenced HUVECs to form vascular structures was not affected by CYD0682. **(S)** CYD0682 significantly promoted the protein expression of ANGPTL4 and inhibited the protein expression of p-p38, p-ERK, and p-JNK. **(T)** CYD0682 did not affect the protein expression of p-p38, p-ERK, and p-JNK in ANGPTL4-silenced HUVECs. ∗*P* < 0.05; ∗∗*P* < 0.01, and ∗∗∗*P* < 0.001.

Given that CYD0682 could significantly inhibit angiogenesis, a breast cancer assay on the CAM was further established to determine whether CYD0682 directly exerted its role in tumor-induced angiogenesis. The vascular plexus of breast cancer on the CAM treated with DMSO, oridonin, or CYD0682 was photographed (Fig. 1E). The tumor volumes and the blood vessel density of oridonin- and CYD0682-treated transplanted breast cancer were significantly smaller and lower than those of DMSO-treated mice (Fig. 1H, I). Importantly, the tumor volumes and the blood vessel density in the CYD0682-treated breast cancer on CAM were significantly decreased compared with those in the oridonin-treated breast cancer, demonstrating that CYD0682 had a stronger ability to inhibit tumor growth and tumor-induced angiogenesis than oridonin (Fig. 1H, I). These results show that CYD0682 significantly inhibits breast cancer growth on CAM by directly attenuating tumor-induced angiogenesis.

Since CYD0682 was found to inhibit angiogenesis, we speculated that CYD0682 may impair tumor-induced angiogenesis, thereby attenuating triple-negative breast cancer growth and metastasis. To define this, the MMTV-PyMT transgenic mice and the MDA-MB-231 xenograft tumor model in nude mice were used to confirm the effect of CYD0682 on triple-negative breast cancer growth and metastasis. The tumor volumes of oridonin- or CYD0682-treated MMTV-PyMT mice (Fig. 1J) and xenograft tumor mice (Fig. 1M) were significantly smaller than those of DMSO-treated mice at the corresponding times, while the tumor volumes of CYD0682-treated mice were significantly decreased compared with those of oridonin-treated mice (Fig. 1J, M). The tumor weights in oridonin- or CYD0682-treated xenograft tumor mice were lighter than those in the DMSO group, while the tumor weights of CYD0682-treated mice were significantly lighter than those of oridonin-treated mice (Fig. 1N). The number of metastatic foci on the lung surface of CYD0682-treated MMTV-PyMT mice was significantly fewer than that in mice treated with DMSO (Fig. 1K). The results of immunohistochemical analysis of CD31 demonstrated that CYD0682 inhibited angiogenesis in MMTV-PyMT mice (Fig. 1L) and xenograft tumor mice (Fig. 1O). Furthermore, we found that CYD0682 had no significant side effects on the histological structure of the heart, liver, lung, and kidney in MMTV-PyMT mice and nude mice (Fig. S2). These data indicate that CYD0682 impedes breast cancer growth and metastasis by attenuating triple-negative breast cancer-induced angiogenesis and has a stronger ability to inhibit breast cancer growth and metastasis than oridonin.

We further investigated whether CYD0682 affected human umbilical vein endothelial cell (HUVEC) proliferation, migration, and tube formation. We found that oridonin and CYD0682 significantly suppressed HUVEC proliferation and migration and the formation of spider-like microvascular capillaries (Fig. 1P), and the images were taken (Fig. S3). To determine the mechanism of CYD0682 in angiogenesis, a quantitative reverse-transcription PCR array was performed to screen for angiogenesis-related genes associated with CYD0682. CYD0682 markedly induced ANGPTL4 expression, and ANGPTL4 expression was found to be significantly increased in HUVECs treated with CYD0682 (Fig. 1Q). HUVEC tube formation significantly increased after silencing ANGPTL4 expression (Fig. 1R). HUVEC vascular structure formation was rarely affected by CYD0682 after ANGPTL4 expression was silenced (Fig. 1R; Fig. S4). Further research demonstrated that CYD0682 increased ANGPTL4 protein expression and inhibited the protein expression of p-p38, p-ERK, and p-JNK in HUVECs (Fig. 1S). As expected, silencing ANGPTL4 inhibited the protein expression of p-p38, p-ERK, and p-JNK (Fig. 1T), while CYD0682 did not change the protein expression of p-p38, p-ERK, and p-JNK after ANGPTL4 was silenced (Fig. 1T). These results suggest that regulating ANGPTL4/mitogen-activated protein kinase (MAPK) signaling pathway is one of the important targets of CYD0682.

Overall, this study has demonstrated that CYD0682, an oridonin analogue, is capable of attenuating breast cancer growth and metastasis in xenograft and transgenic mouse models. Importantly, CYD0682 exhibits a better inhibitory effect on breast cancer growth than oridonin. Its inhibitory effects appear to be attributed to suppressing tumor-induced angiogenesis. Furthermore, regulating the ANGPTL4/MAPK signaling pathway has been determined as one dominant antiangiogenic signaling of CYD0682. Our results support that CYD0682 may have great potential to be developed as a promising anti-cancer agent for the treatment of breast cancer, particularly the aggressive triple-negative breast cancer.

## CRediT authorship contribution statement

**Xiaobin Mai:** Methodology, Formal analysis, Data curation. **Le Wang:** Methodology, Formal analysis, Data curation. **Juan Tu:** Methodology, Formal analysis, Data curation. **Jialin Li:** Methodology, Formal analysis, Data curation. **Jun Li:** Methodology. **Yaping Zhan:** Methodology, Data curation. **Pei Tang:** Supervision, Project administration, Funding acquisition. **Ying Wang:** Supervision, Methodology, Data curation. **Yan Wang:** Methodology, Data curation. **Lingyun Zheng:** Supervision. **Qianqian Zhang:** Supervision. **Jiangchao Li:** Supervision. **Xiong Li:** Supervision. **Lijing Wang:** Supervision, Data curation, Conceptualization. **Jia Zhou:** Writing – review & editing, Supervision, Data curation, Conceptualization. **Cuiling Qi:** Writing – review & editing, Writing – original draft, Supervision, Data curation, Conceptualization.

## Ethics declaration

All animal experiments were conducted according to relevant national and international guidelines. All the animal procedures were approved by the Medical Research Animal Ethics Committee of Guangdong Pharmaceutical University.

## Funding

This work was supported by the Basic and Applied Basic Research Project of Guangdong Province, China (No. 2023A1515010459 to C.L.Q.), Key Team of Basic and Clinical Research on Tumor Immunotherapy of Guangdong Pharmaceutical University (No. 2024ZZ10 to X.L.), and the Project of Administration of Traditional Chinese Medicine of Guangdong Province, China (No. 20251209 to C.L.Q.; 20242048 to P.T.). Dr. Jia Zhou has no connections with the above-mentioned funding resources in China, and is partly supported by the John D. Stobo, M.D. Distinguished Chair Endowment Fund, and Edith & Robert Zinn Chair in Drug Discovery Endowment Fund at the 10.13039/100007865University of Texas Medical Branch in the United States.

## Conflict of interests

Dr. Jia Zhou is an Associate Editor of the journal *Genes & Diseases*. The other authors declare no potential conflict of interests.
