# Supplementary material for: A novel oridonin analogue, CYD0682, suppresses breast cancer growth, angiogenesis, and metastasis by inhibiting the ANGPTL4/MAPK signaling axis
Source: Genes Dis. 2025 May 8;12(5):101681. doi: 10.1016/j.gendis.2025.101681 (PMC12166989; doi:10.1016/j.gendis.2025.101681)
Supplement: Multimedia component 1 [file mmc1.docx]

**Supplementary Information**

**A novel oridonin analogue, CYD0682, suppresses breast cancer growth, angiogenesis and metastasis by inhibiting ANGPTL4/MAPK signaling axis**

Xiaobin Mai^a,1^, Le Wang^a,1^, Juan Tu^a,1^, Jialin Li^a,1^, Jun Li^b^, Yaping Zhan^a^, Pei Tang^a^, Ying Wang^a^, Yan Wang^a^, Lingyun Zheng^a^, Qianqian Zhang^a^, Jiangchao Li^a^, Xiong Li^a^, Lijing Wang^a,^*, Jia Zhou^b,^**, Cuiling Qi^a,^*

^1^ These authors contributed equally to this work.

*Corresponding authors. *E-mail addresses:* qicuiling12345@163.com (C.L. Qi), [jizhou@utmb.edu](mailto:jizhou@utmb.edu.) (J. Zhou), [wanglijing62@163.com](mailto:wanglijing62@163.com) (L.J. Wang)

**This file includes:**

Supplemental Table S1

Supplemental Fig. S1 to S4

Methods and Materials

Abbreviations

**Table S1.** Screening identified CYD0682 as a potent antiangiogenic drug. The oridonin analogues were added onto chick embryo CAM. Preliminarily screen identified four compounds that could potently suppress angiogenesis. YSM assay was further used to evaluate these compounds, and CYD0682 was identified as the most effective agent.

| Methods | Potentially antiangiogenic compounds identified |
| --- | --- |
| CAM | CYD0281, CYD0682, CYD0628, and CYD0907 |
| YSM | **CYD0628** |

**Supplementary Fig. S1 to S4**


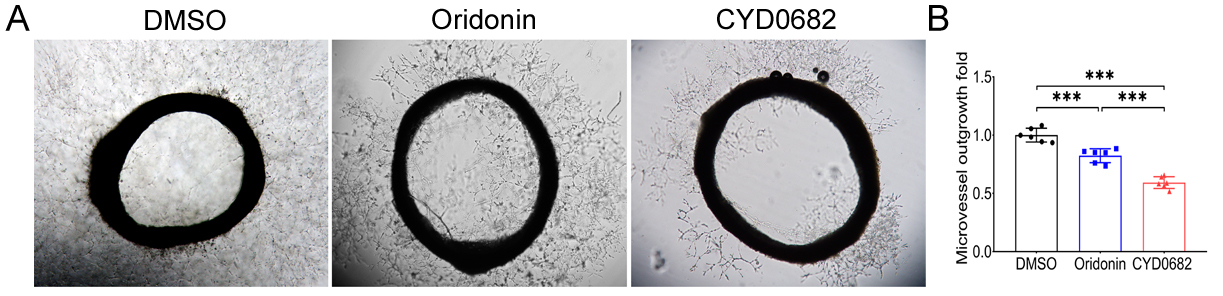


**Figure S1.** The effects of CYD0682 on microvessel outgrowth of the aortic rings. (**A)** Representative pictures of sprouting from aortic rings treated with DMSO or oridonin or CYD0682. (**B)** CYD0682 significantly inhibited the microvessel outgrowth from aortic rings.


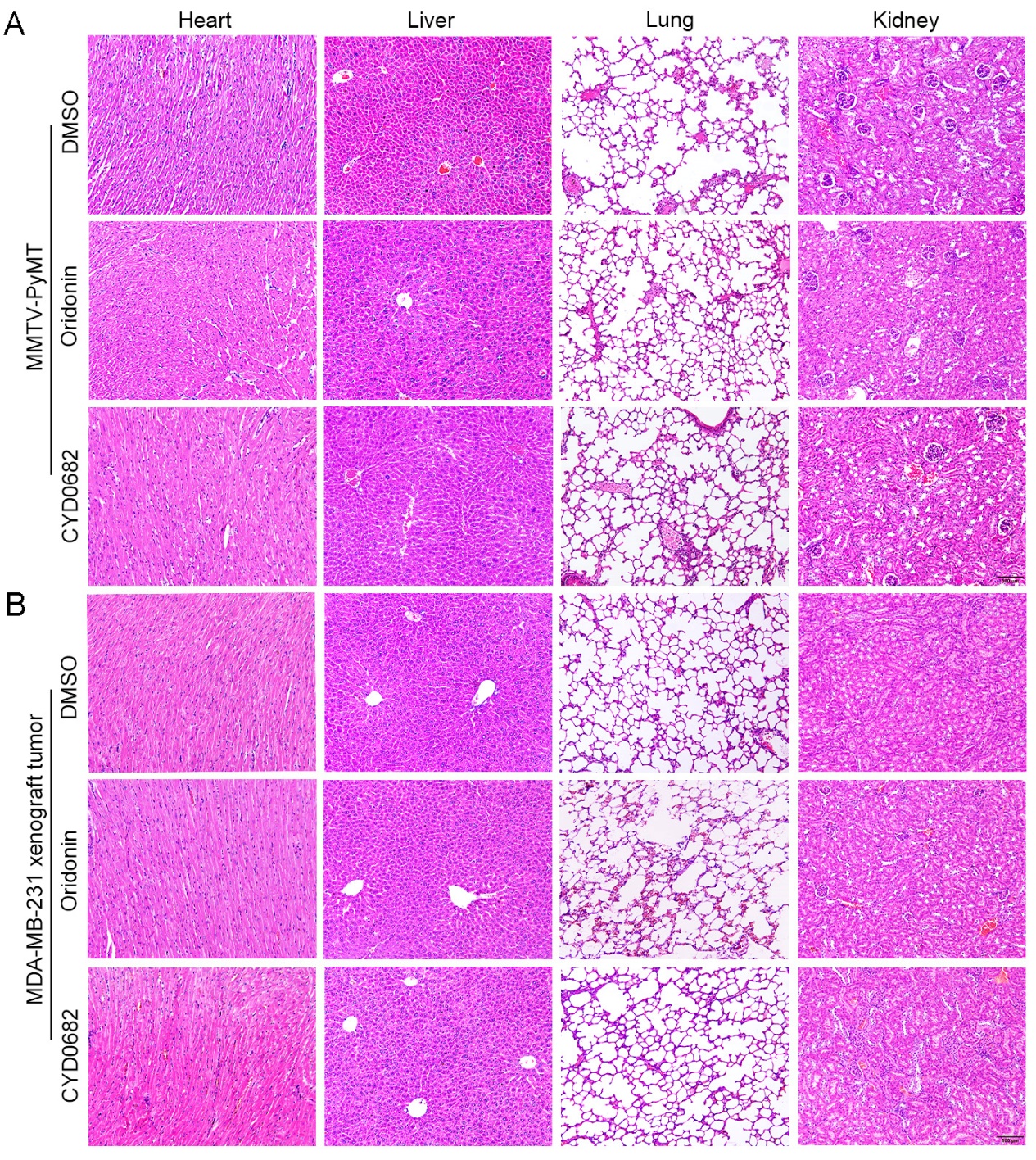


**Figure S2.** The effects of DMSO, CYD0682 or oridonin on histological structures of heart, liver, lung and kidney in the MMTV-PyMT mice and transplanted tumor mice. **(A, B)** The histological structure of heart, liver, lung and kidney did not change after the mice were treated with DMSO, CYD0682 or oridonin. Scarl bar = 100 µM.


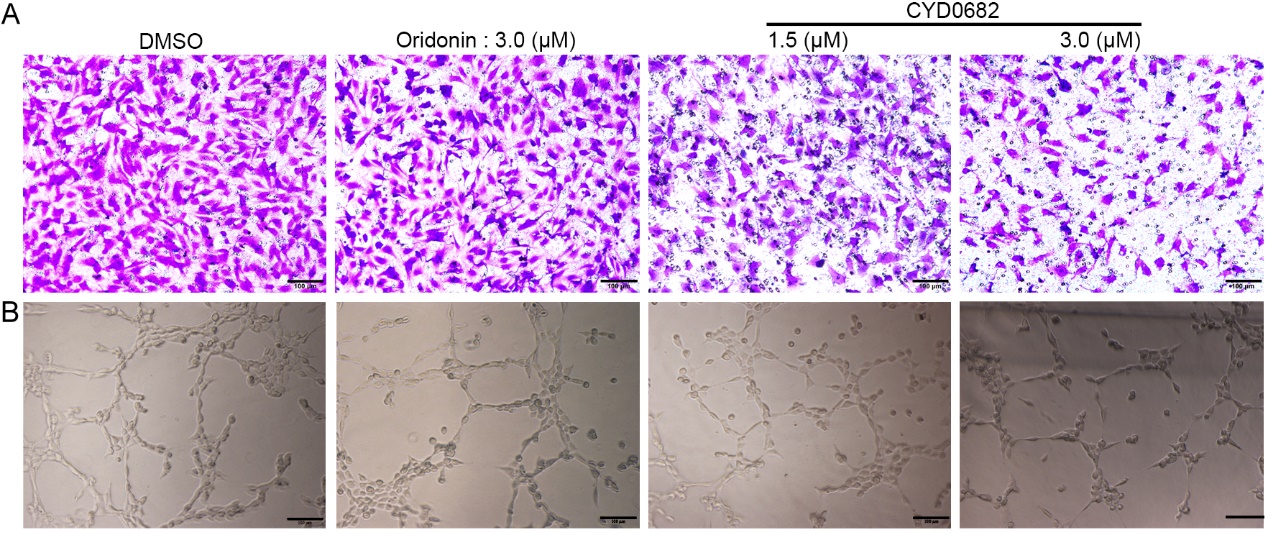


**Figure S3.** CYD0682 suppressed HUVEC migration and tube formation. **(A)** The migration ability of CYD0682 on HUVECs was assessed using a transwell assays. HUVECs were treated with DMSO or oridonin (3 μM) or CYD0682 (1.5 μM or 3 μM). Representative images of migrated HUVECs were photographed, and the number was counted. **(B)** The effect of CYD0682 on capillary-like tube formation was calculated using tube formation assay. Representative images of tube formation were photographed. Scale bars = 100 μm in A and B.


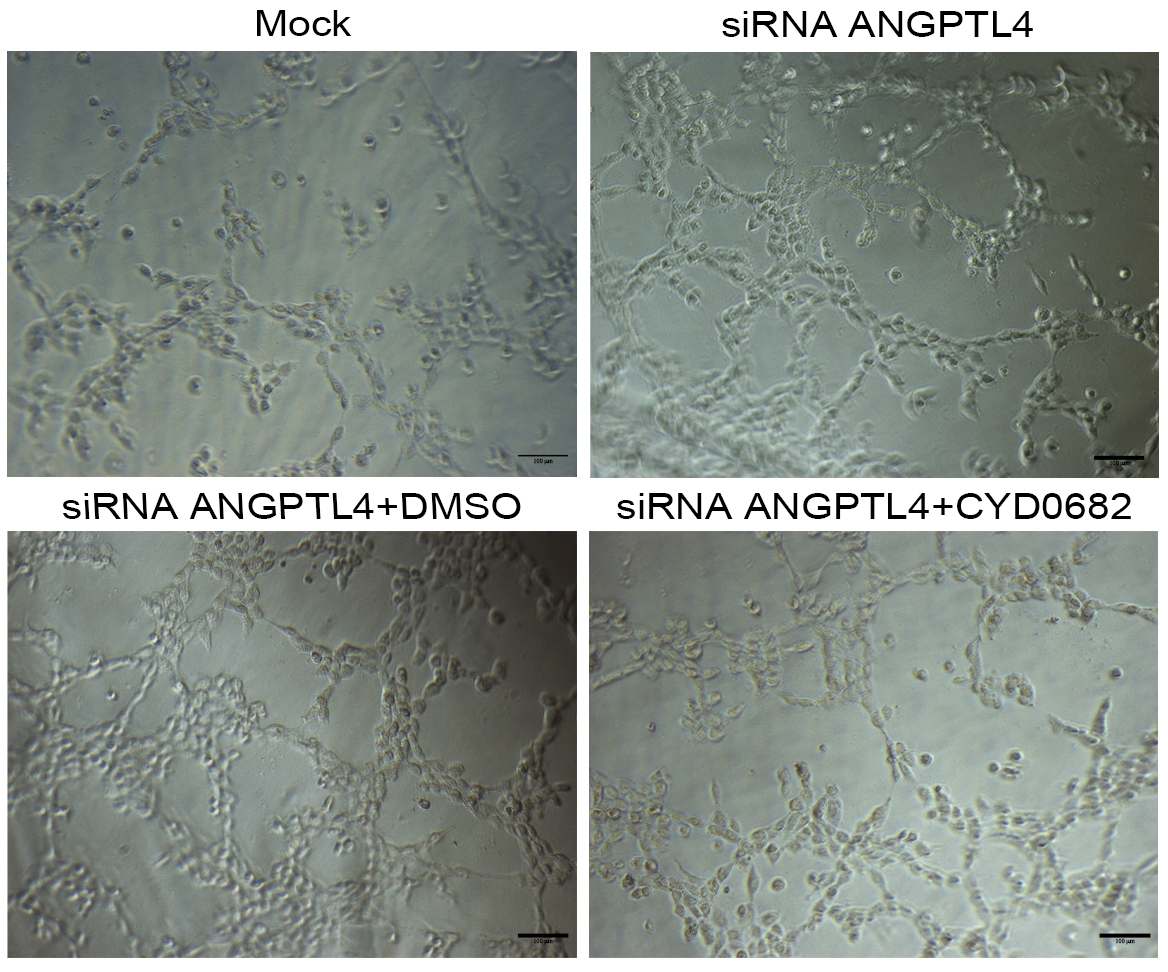


**Figure S4.** CYD0682 suppressed angiogenesis through up-regulating ANGPTL4 expression. The effect of CYD0682 on capillary-like tube formation was calculated using tube formation assay after ANGPTL4 was silenced. Representative images of tube formation were photographed. Scale bars = 100 μm.

**Materials and methods**

**Chick embryo chorioallantoic membrane (CAM) assay**

The CAM model was utilized to explore *in vivo* angiogenic activity as we previously established.^1^ Briefly, 7-day-old fertilized chicken eggs were used to perform the CAM assay. After being incubated at 38 °C and 36.5~38.5% humidity for 48 hours, chicken eggs were opened a window in the eggshell. The sterilized silicone ring was put on the primary or secondary blood vessels of the CAM. Either 0.5 and 1.0 µg of CYD0682 or 1.0 µg oridonin (Sigma-Aldrich, St Louis, MO, USA) or DMSO (Sigma-Aldrich, St Louis, MO, USA) were added into the ring on the CAM of 9-day-old chicken embryos, and then the embryos were incubated for 48 hours. The pictures of the vascular plexus of the CAM were taken and the effects of CYD0682 on angiogenesis were calculated through assessing the blood vessel density using the image analysis software IPP 6.0 (Image Pro-Plus, version 6.0, Media Cybernetics).

**Chick embryo yolk sac membrane (YSM) assay**

The YSM assay was utilized to further explore the effect of CYD0682 on angiogenesis as previously described.^1^ Four-day-old fertilized chicken eggs were placed into the sterilized dishes, and then two rings were symmetrically put on the blood vessel of the YSM. Either 0.5 and 1.0 µg of CYD0682 or 1.0 µg oridonin or DMSO (Sigma-Aldrich, St Louis, MO, USA) were added into the ring on the vascular network of the YSM. The images of the blood vessels within the rings were taken at 0 h, 12 h and 24 h after treatment. The blood vessel density in the pictures was calculated using the image analysis software IPP 6.0.

**High-throughput screening**

The compound library of the oridonin analogues, which was synthesized by Professor Zhou's team following a patent filed by UTMB, was utilized to screen for the compounds with higher anti-cancer efficacy and lower toxicity. All new compounds were validated by NMRs and HPLC analysis (purity >98%), and then delivered to Guangdong Pharmaceutical University according to the mutually signed material transfer agreement (MTA) for further biological testing, which was approved by the Office of Technology Transfer (OTT) of the UTMB. 2 µg oridonin analogue or DMSO was added onto the CAM of 9-day-old embryos and incubated for 48 h. After incubation, the potential anti-angiogenic activity of the oridonin analogues were preliminarily assessed. YSM model was further used to identify the promising anti-angiogenic agents from the selected active compounds.

**Rat aortic ring assay**

The rat aortic ring assay was carried out as previously described by us.^2^ Briefly, the thoracic aortas were isolated from 9-week-old male Sprague-Dawley (SD) rats, and then were cut into 1 - 2 mm long rings, which were put into 48-well plates coated with 100 μL Matrigel. After the Matrigel was solidified, 200 μL of EBM-2 medium (cat. no. CC-2935, LONZA, Walkersville, MD, USA) containing oridonin or CYD0682 or DMSO was added to the wells of 48-well plates and incubated at 37 °C in 5% CO_2_ for 7 days. After fixation with 4% formalin, vessel sprouting of aortic ring were taken using the inverted microscope. Next, analysis of the microvessel growth was performed by counting the number of the blood vessels using the image analysis software IPP 6.0.

**MDA-MB-231 breast cancer assay on CAM**

To explore whether CYD0682 has a direct inhibitory effect on angiogenesis, MDA-MB-231 breast cancer xenograft tumor model on CAM was established as previously described.^3^ Briefly, 8-day-old chicken embryos were windowed on the shell above the air chamber, and then a silastic ring was put on the blood vessel of the CAM. MDA-MB-231 cells (1 × 10^7^/50 µL) were loaded in the rings. Two days later, 30 μL of oridonin (1 μg), CYD0682 (0.5 and 1 μg), or DMSO was added into the silastic rings, and then the chicken embryos were incubated at 37 °C. Two days later, the eggs were cut open along the axis of the median line and the blood vessels of the breast tumor on CAM were photographed and calculated using the image analysis software IPP 6.0. The length and width of the breast tumors were measured and the tumor volumes were estimated according to the following formula: 0.52 × length × width^2^.

**MMTV-PyMT spontaneous breast cancer model and CYD0682 treatment**

Oridonin (7.5 mg/kg) or CYD0682 (7.5 mg/kg) or DMSO were intraperitoneally injected into 9-week-old MMTV-PyMT mice once every 2 days for 4 weeks. The length and width of the breast tumors were measured every 3 days, and the tumor volumes were assessed according to the following formula: 0.52 × length × width^2^.^4^ The MMTV-PyMT mice were euthanized after 30 days, and then the tumors, lungs, hearts, kidneys and livers were isolated to weigh the tumors, record the number of pulmonary metastatic foci and perform histological analysis.

**MDA-MB-231 subcutaneous xenograft tumor model and CYD0682 treatment**

For the xenograft breast cancer model, 1×10^6^ MDA-MB-231 cells were injected subcutaneously into the mammary fat pads of each 6-week-old male athymic nude mice. When the tumors were visible, DMSO or oridonin (7.5 mg/kg, positive control) or CYD0682 (7.5 mg/kg) was intraperitoneally injected into the athymic nude mice every day for up to 20 days. Breast cancer growth was monitored by measuring tumor length and width every 2 days, and the tumor volume was assessed according to the following formula: 0.52 × length × width^2^. The tumor-bearing mice were euthanized after 20 days, and the tumors, lungs, hearts, kidneys and livers were isolated for analysis.

**Hematoxylin-eosin (H&E) staining**

H&E staining was performed to observe the changes in the tissue structure of important organs in mice treated with CYD0682. The lungs, hearts, kidneys and livers were fixed in formalin, embedded and sectioned. H&E staining was performed on the paraffin-embedded sections (3 μm thick). After dewaxed and hydrated, the tissue sections were stained with hematoxylin and eosin according to standard protocols. The tissue structures were evaluated independently by two observers.

**Immunohistological (IHC) staining**

The tumor tissues were fixed in formalin for at least 24 h, embedded and sectioned. Immunohistochemical staining was performed on the paraffin-embedded sections (6 μm thick). After dewaxed and hydrated, the tissue sections were incubated with anti-CD31 (cat. no. ab28364, Abcam, Cambridge, CB, UK) or anti-Ki67 (cat. no. ab279653, Abcam) primary antibodies at 4 ℃ overnight. The next day, the tissue sections were incubated with HRP-conjugated secondary antibodies, stained with DAB and counterstained with hematoxylin. The number of CD31^+^ vessels in a 200 × field were counted as the density of microvessels.^5^ The Ki67^+^ cells were counted in a 400× field, and the percentage of Ki67^+^ cells to the total cells was considered as the proliferative index[31]. All the sections were evaluated by two experimenters.

**MTT assay**

MTT assay was explored to calculate the effect of CYD0682 on human umbilical vein endothelial cells (HUVECs) or breast cancer cell proliferation. HUVECs (5 × 10^3^/well) or human breast cancer MDA-MB-231 cells (3 × 10^3^/well) were added into 96 well plates and then treated with oridonin or CYD0682 or DMSO. After treatment for 48 h, 5 mg/mL MTT solution was added to each well. The plates were incubated at 37 ºC for 4 h, and then the supernatant was removed and 150 µL of DMSO was added. After the resultant formazan crystals were dissolved by DMSO, the OD value was measured at 490 nm and 570 nm using microplate reader. The IC_50_ value was assessed using Graph8.0 software.

**Cell migration assay**

Cell migration assay was used to evaluate the ability of CYD0682 to inhibit HUVEC or breast cancer cell migration. HUVECs or MDA-MB-231 cells were harvested and suspended in serum-free media containing oridonin (3 µM) or different concentrations of CYD0682 (1.5 µM or 3 µM) or the corresponding dose of DMSO. HUVECs (2 × 10^4^ cells/200 µL) or MDA-MB-231 cells (4 × 10^4^ cells/200 µL) were added to the upper chambers of the transwells and the lower chambers were added EBM containing 20% FBS or DMEM medium containing 50% FBS, respectively. After HUVECs or MDA-MB-231 cells were incubated for 12 h or 10 h, the cells on the upper side of the membrane were wiped off using a cotton swab. After fixed in 4% paraformaldehyde for 30 min, the cells were stained with 1% crystal violet for 20 min. The number of migratory cells was quantified using the image analysis software IPP 6.0.

**Transwell invasion assay**

Cell invasion assay was used to evaluate the ability of CYD0682 to inhibit breast cancer cell invasion. Human breast cancer MDA-MB-231 cells were suspended in serum-free media containing oridonin (3 µM) or different concentrations of CYD0682 (1.5 µM or 3 µM) or the corresponding dose of DMSO. MDA-MB-231 cells (6 × 10^4^ cells/200 µL) were introduced to the upper chambers of the transwells pre-coated with Matrigel (BD Biosciences), and EBM containing 50% FBS were added into the lower chambers. After MDA-MB-231 cells were incubated for 24 h, the cells were fixed in 4% paraformaldehyde for 30 min and stained with 1% crystal violet for 20 min. The result was analyzed by the image analysis software IPP 6.0.

**Colony formation assay**

MDA-MB-231 cells (1 × 10^3^/well) were seeded onto 6-well plates and treated with CYD0682 or oridonin or DMSO for 7 days. After being fixed with 4% paraformaldehyde for 30 min, the colonies were stained with 1% crystal violet solution for 20 min. The colonies were photograthed and the number of the colonies was counted by the image analysis software IPP 6.0. The experiment was repeated three times.

**Tube formation assay**

The effect of CYD0682 on capillary-like tube formation was calculated using HUVECs. HUVECs (1.5 × 10^5^ cells/mL) treated with oridonin or CYD0682 or DMSO or ANGPT4 siRNA were uniformly distributed over 96-well chambers, which were coated with 30 µL of growth factor-reduced Matrigel (cat. no. 356230, BD Biosciences, Becton Dickinson, San Jose, CA). Next, the presence of tube-like structures was photographed using the inverted microscope after the cells were incubated at 37 °C for 3-5 h. The total tube length was determined using IPP software (Media Cybernetics).

**Quantitative real-time PCR (qRT-PCR)**

The total RNA was extracted from the HUVECs after the cells were treated with CYD0682 (3 µM) or DMSO for 48 h. A qRT-PCR array about angiogenesis-related genes (cat. no. PAMM-024A, SA Biosciences) was used to screen CYD0682-related genes. The screened genes were further determined using total RNA extracted from HUVECs and tumor tissues, which were treated with CYD0682 or DMSO. Triplicate qRT-PCR experiments were explored for each of the samples analyzed.

**Western blotting**

After treated with CYD0682 (3 μM) or DMSO for 48 h, HUVECs and MDA-MB-231 cells were lysed using RIPA lysis buffer (Thermo Scientific, Scotts Valley, CA, USA). After quantified using BCA protein assay kit (Boster Bio Tech, Wuhan, China), the proteins were separated by SDS-polyacrylamide gels and then electroblotted onto PVDF membranes (Millipore, Billerica, MA, USA). The membranes were blocked in skim milk and incubated with primary antibodies at 4 °C overnight. The primary antibodies used here included: anti-ANGPT4 (cat. no. A01147, BOSTER), anti-phospho-p38 (cat. no. 4511S, Cell Signaling Technology), anti-p38 (cat. no. 8690S), anti-phospho-ERK (cat. no.9101S, Cell Signaling Technology), anti-ERK (cat. no.9102S, Cell Signaling Technology), anti-phospho-JNK (cat. no.9255S, Cell Signaling Technology), anti-JNK (cat. no.9252S, Cell Signaling Technology), anti-GAPDH (cat. no.2118S, Cell Signaling Technology). Next, the membranes were incubated with horseradish peroxidase-coupled IgG for 1 hour at room temperature. The signal was detected using an Odyssey Infrared Imager (LICOR Bioscience, Lincoln, NE, USA).

**Statistical analysis**

The GraphPad Prism 8.0 software package (GraphPad Software, CA) were used to analyze the data and construct the statistical charts. For statistical analysis, the paired two-tailed Student’s t-test was employed to determine the statistical significance between two groups, and the one-factor analysis of variance (ANOVA)was used to determine the statistical significance between multiple groups. The differences were considered significant at *p* < 0.05.

**Supplemental References**

1. Cao JH, Liu XH, Yang Y, et al. Decylubiquinone suppresses breast cancer growth and metastasis by inhibiting angiogenesis via the ROS/p53/BAI1 signaling pathway. *Angiogenesis*. 2020;23(3):325-338.

2. Han BA, Zhang H, Tian RN, et al. Exosomal EPHA2 derived from highly metastatic breast cancer cells promotes angiogenesis by activating the AMPK signaling pathway through Ephrin A1-EPHA2 forward signaling. *Theranostics.* 2022;12(9):4127-4146.

3. Miebach L, Berner JL, Bekeschus S, et al. *In ovo* model in cancer research and tumor immunology. [*Front Immunol.*](https://www.ncbi.nlm.nih.gov/pmc/articles/PMC9556724/) 2022;13:1006064.

4. Li JL, Zheng ST, Cheng T, et al. Decylubiquinone inhibits colorectal cancer growth through upregulating sirtuin2. [*Front Pharmacol.*](https://www.ncbi.nlm.nih.gov/pmc/articles/PMC8844026/) 2021;12:804265.

5. Zheng ZQ, Chen JT, Zheng MC, et al. Nestin^+^/CD31^+^ cells in the hypoxic perivascular niche regulate glioblastoma chemoresistance by upregulating JAG1 and DLL4. *Neuro Oncol.* 2021; 23(6):905-919.

**Abbreviations**

CAM: Chick embryo chorioallantoic membrane; YSM: Yolk sac membrane; HUVEC: Human endothelial cell; ANGPTL4: Angiopoietin-like protein 4; LPL: Lipoprotein lipase; H&E: Hematoxylin-eosin; qRT-PCR: Quantitative real-time PCR; IHC: Immunohistological staining; DAB: Diaminobenzidine; MVD: Micro-vascular density
